# Should Not the Increase of Dental Schools Be Restricted?

**Published:** 1895-12

**Authors:** Louis Jack

**Affiliations:** Philadelphia


### Should not the Increase of Dental Schools be Restricted?

BY LOUIS JACK, D.D.S., PHILADELPHIA.

Abstract of paper read at American Dental Association, Aug., 1895.

In this paper the writer referred to the rules passed by the Faculties' Association regarding the requirements of Colleges applying for membership in that body. He said that the tendency to rapid increase of dental schools is shown by the statistics since 1891. In 1893 there were eight unrecognized schools; in 1894, fifteen. Of these, three have been recognized this year, making thirty-one recognized schools, while there are now eighteen unrecognized. The diploma of an unrecognized school has a legal value in the State where the school is located; it gives no *prima facie* right to practice elsewhere without examination by the state board, and in some States the holder would not have the privilege of being examined.

He spoke of the prevailing mania for forming dental schools in connection with medical colleges and pointed out the dangers of over-production that might be brought about unless some restrictions were made. One prominent difference between dental colleges in connection with medical schools and those in universities was that the latter were founded on a broader basis.

Unless some brake is put upon the addition of dental schools which are not of high order, the prejudice against the dental institutions of this country must further increase. The States have no principle which can be exercised to regulate the quantity of schools. We have to depend upon the development of opinion to assist and support such bodies as the Faculty Association or the Dental Examiners, in the formulation of such rules as shall guard the interests of all concerned, and to aid in checking the degradation of a profession.

